# The pitfalls of interpreting hyperintense FLAIR signal as lymph outside the human brain

**DOI:** 10.1038/s41467-023-40508-2

**Published:** 2023-08-16

**Authors:** Geir Ringstad, Per Kristian Eide

**Affiliations:** 1 Department of Radiology and Nuclear Medicine, Oslo University Hospital - Rikshospitalet, Oslo, Norway; 2 Department of Geriatrics and Internal Medicine, Sorlandet Hospital, Arendal, Norway; 3 Department of Neurosurgery, Oslo University Hospital-Rikshospitalet, Oslo, Norway; 4 Institute of Clinical Medicine, Faculty of Medicine, University of Oslo, Oslo, Norway

**Keywords:** Neurology, Neuroscience

**arising from** M. S. Albayram et al. *Nature Communications* 10.1038/s41467-021-27887-0 (2022)

Lymphatic vessels within the rodent meninges were in 2015 discovered to drain cerebrospinal fluid (CSF)^1,2^. In a recent research article, Albayram et al.^3^ claims to provide a detailed visualization of human lymphatic structures within the intracranial dura and cervical vessel walls defined by hyperintense (bright) MRI signal at FLAIR (fluid-attenuated inversion recovery) in a variety of locations. Even though a hyperintense FLAIR signal at MRI has many sources, including image artifacts, the authors interpret it all to be lymph.

Tissue with hyperintense FLAIR signal in the parasagittal dura along the superior sagittal sinus was accepted to reflect rodent dorsal meningeal lymphatic vessels, and hyperintense FLAIR signal along cranial nerves and in dura at the skull base was accepted to represent ventral meningeal lymphatic vessels. The authors argue that their findings of continuous FLAIR-signal between intracranial dura and neck vessel walls represent lymphatic CSF drainage pathways from the dura to deep cervical lymph nodes, which are also characterized by hyperintense FLAIR signal. Validation of results was provided from a phantom study where assumed regions of lymphatics at FLAIR corresponded to a protein concentration ranging from 2000 to 4000 mg/dl. Even though this was higher than what has been reported in lymph, they speculate this may be related to “cells, solutes, debris and other proteins”.

We are here at the core of our concern about this study: While the FLAIR sequence at MRI is sensitive to water within a protein-rich environment, it is also highly unspecific. Hyperintense FLAIR signal resembling the FLAIR signal characterized by proteinaceous fluid is no proof of concept for having detected lymph. On the contrary, hyperintense FLAIR signal can occur outside the brain for many reasons. With concern to the parasagittal dura, there has previously been described several elements within this region that can contribute to a hyperintense FLAIR signal. In their anatomical study, Fox et al.^4^ showed contents of arteries and veins, furthermore an extensive network of channels coalescing toward the superior sagittal sinus, possibly carrying CSF, and a “dense carpet” of intradural arachnoid granulations. Kerber and Newton demonstrated a venous plexus in dura that was particularly dense in the parasagittal region^5^, suggested to play a role in CSF absorption. Recently, Shah et al. demonstrated that the hyperintense FLAIR signal in parasagittal dura corresponds to arachnoid granulations embedded within a dural stroma consisting of a collagen and fibronectin-rich tissue, harboring diverse immune cell populations^6^. Park et al. showed in their human study dural channels that may serve as a reservoir for CSF drainage and found at the same time no evidence of dural lymphatic vessels^7^. We recently also confirmed efflux of a CSF tracer to parasagittal dura in a human cohort, but with no evidence of the tracer accumulating in lymph-like vessels detectable at MRI^8^. Proteins and cells derived from the blood pool, intradural arachnoid granulations and CSF thus constitute most plausible sources of increased FLAIR signal in this region. To this end, as initial lymphatic vasculature in parasagittal dura has been shown to have size at micrometer scale, and populate a minor proportion of the tissue stroma^9^, the high signal depicted on FLAIR at scale three orders of magnitude higher (millimeter) is very likely not lymph. The scientific basis for accepting increased FLAIR signal in parasagittal dura as lymph is therefore lacking.

Another well-known source of bright signal at FLAIR images is CSF flow artifacts near the skull base, and these artifacts may be particularly abundant at 3 Tesla^10,11^, the same magnetic field strength that was used in the current study. In their Figure 4 arrows point to what is in the legend denoted as “ventral dural lymphatic system MR images and metrics from multiple subjects” (Fig. 1a). In several of the figure elements arrows point directly at regions of bright FLAIR signal that we, with little doubt, depict as CSF flow artifacts within the subarachnoid fluid space^11^. At our hospital, we have the same 3 Tesla Siemens Prisma scanner (Siemens, Erlangen) as in the current study, and even with use of techniques to compensate for flow artifacts, we struggle with artifacts underneath the brain very similar to the ones Albayram et al.^3^ have pointed out as representations of ventral dural lymphatics (see examples provided in Fig. 1).

**Fig. 1:**
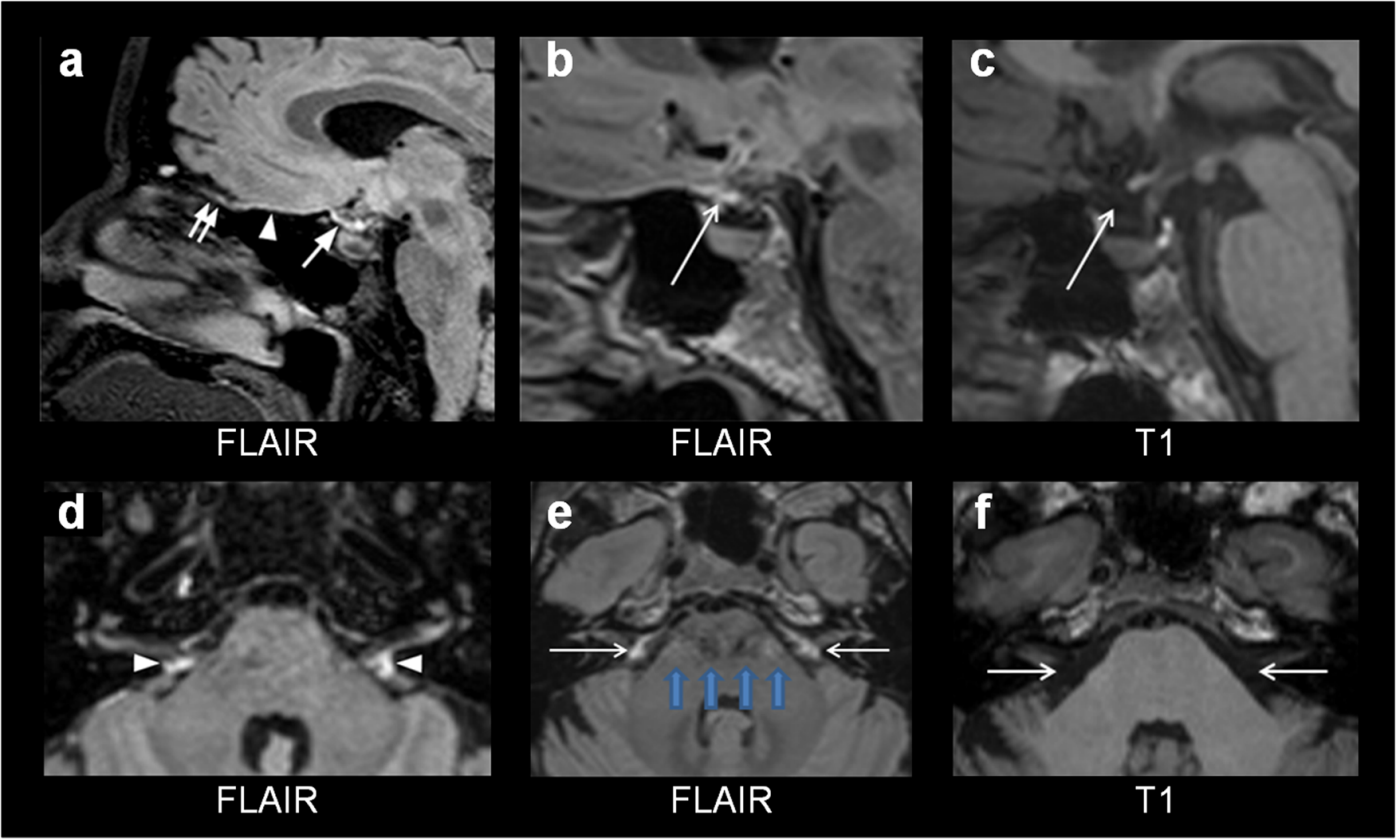
CSF flow artifacts at FLAIR in the subarachnoid space. **a** In their Figure 4a, Albayram et al.^3^ point at a linear hyperintensity at sagittal FLAIR in the suprasellar cistern and identify this as lymph. **b** Image from our same Prisma MRI scanner (Siemens, Erlangen) shows a similar phenomenon (arrow). **c** At T1 weighted MRI through the same section it becomes clear that the FLAIR hyperintensity is within the CSF compartment (arrows), not the dura itself, which in this region is constituted by the sellar diaphragm immediately adjacent to the upper border of the pituitary gland. This clearly indicates a flow artifact derived from CSF pulsations within the suprasellar cistern. **d** In their Figure 4f, Albayram et al.^3^ point at FLAIR hyperintensities (arrowheads) within the cerebellopontine angle cisternal spaces and identify them as lymph (axial section). **e** We reproduce similar findings (arrows) at our same MRI Prisma scanner (Siemens, Erlangen). Propagation of artifacts through the brain stem (blue arrows) in the same level (along the phase encoding direction), as also seen to a minor extent in **d**, is highly suggestive that these are CSF flow artifacts. **f** At T1 weighted MRI through the same imaging plane it becomes clear that the hyperintensities at FLAIR correspond to the CSF space (arrows), not the dura or cranial nerves.

In the neck soft tissue, it appears that the authors have interpreted almost any structure with hyperintense FLAIR signal to be lymph. The smooth, continuous FLAIR signal defining the wall of the jugular vein is in Figure 5 by Albayram et al.^3^ interpreted as “prominent lymphatic fluid signal”. It is unclear what the scientific foundation behind this assumption is. Rodent studies have up to recently demonstrated lymphatic CSF drainage along the carotid arteries^12^, not in venous walls. While animal^2^ and human^13^ studies have yet to directly visualize connections between neck lymphatic vessels and neck lymph nodes, the authors claim to reveal “lymphatic connections from the cranial nerve IX–XI complex to the deep cervical lymph nodes” by their identification of subtle linear structures of FLAIR hyperintensity. Why this could not represent for instance slow flow within small veins, is not discussed.

We acknowledge the importance of studies attempting to translate findings from basic to clinical medicine. A better understanding of human dural lymphatics and meningeal immunity can have large implications for how we approach neurological disease. However, translational imaging studies are only meaningful when findings made in humans are critically assessed on basis of an in-depth knowledge of preceding animal studies. A further prerequisite is insight into inherent limitations of the applied imaging techniques, which should be expected with well-established methods like FLAIR^14^. In the current study, conclusions have unfortunately been drawn quite prematurely about the ability of FLAIR to detect lymph at the parasagittal dura, the skull base, and at the neck. When Albayram et al.^3^ conclude that “this non-invasive technique could be used to evaluate the MVLs system and function of the brain”, we are concerned this will prepare the ground for further research efforts based on similarly flawed conditions.

## Methods

The MRI shown in Fig. 1 was obtained as part of a study of patients undergoing diagnostic work-up of epilepsy, approved by The Regional Committee for Medical and Health Research Ethics (REK) of Health Region South-East, Norway (2020/121533), and was obtained after written and oral informed consent. We used a 3 Siemens Prisma MRI scanner with sequence parameters including the following:

1. 3D T1-gradient echo (T1-MPRAGE-ADNI): sagittal scanning plane; repetition time (TR) = 2300 ms; echo time (TE) = 2.93 ms; inversion time = 900 ms flip angle = 9°; 1 average; 1 mm isotropic voxels.
2. 3D T2-Fluid Attenuated Inversion Recovery (FLAIR): sagittal scanning plane; TR = 5000 ms; TE = 386 ms; inversion time = 1800 ms; flip angle = 120°; 1 average; 0.9 mm isotropic voxels

### Reporting summary

Further information on research design is available in the Nature Portfolio Reporting Summary linked to this article.

### Supplementary information

Reporting Summary

## Data Availability

The MRI acquisitions shown in Fig. 1 are not publicly available since they represent patient-sensitive information. The data will be made available through requests to the corresponding author for a time of 6 months and will require a data use agreement for access.
